# Young pregnant women and risk for mental disorders: findings from an early pregnancy cohort

**DOI:** 10.1192/bjo.2019.6

**Published:** 2019-03-07

**Authors:** Georgia Lockwood Estrin, Elizabeth G. Ryan, Kylee Trevillion, Jill Demilew, Debra Bick, Andrew Pickles, Louise Michele Howard

**Affiliations:** Senior Research Associate, Section of Women's Mental Health, Institute of Psychiatry, Psychology and Neuroscience, King's College London; and Research Fellow, Centre for Brain and Cognitive Development, Birkbeck College, UK; Senior Research Fellow, Biostatistics and Health Informatics Department, Institute of Psychiatry, Psychology and Neuroscience, King's College London; and Clinical Trials Unit, Warwick Medical School, University of Warwick, UK; Lecturer, Section of Women's Mental Health, Institute of Psychiatry, Psychology and Neuroscience, King's College London, UK; Consultant Midwife, Women's Health, King's College Hospital NHS Foundation Trust, UK; Department of Women and Children's Health, School of Life Course Sciences, Faculty of Life Sciences and Medicine, King's College London; and Professor of Clinical Trials in Maternal Health, Warwick Clinical Trials Unit, Warwick Medical School, University of Warwick, UK; Chair in Biostatistics, Biostatistics and Health Informatics Department, Institute of Psychiatry, Psychology and Neuroscience, King's College London, UK; Professor in Women's Mental Health and Head of the Section of Women's Mental Health, Section of Women's Mental Health, Institute of Psychiatry, Psychology and Neuroscience, King's College London; Department of Women and Children's Health, School of Life Course Sciences, Faculty of Life Sciences and Medicine, King's College London; and South London and Maudsley NHS Foundation Trust, UK

**Keywords:** Trauma, anxiety disorders, perinatal psychiatry, depressive disorders

## Abstract

**Background:**

Young women aged 16–24 are at high risk of common mental disorders (CMDs), but the risk during pregnancy is unclear.

**Aims:**

To compare the population prevalence of CMDs in pregnant women aged 16–24 with pregnant women ≥25 years in a representative cohort, hypothesising that younger women are at higher risk of CMDs (depression, anxiety disorders, post-traumatic stress disorder, obsessive–compulsive disorder), and that this is associated with low social support, higher rates of lifetime abuse and unemployment.

**Method:**

Analysis of cross-sectional baseline data from a cohort of 545 women (of whom 57 were aged 16–24 years), attending a South London maternity service, with recruitment stratified by endorsement of questions on low mood, interviewed with the Structured Clinical Interview DSM-IV-TR.

**Results:**

Population prevalence estimates of CMDs were 45.1% (95% CI 23.5–68.7) in young women and 15.5% (95% CI 12.0–19.8) in women ≥25, and for ‘any mental disorder’ 67.2% (95% CI 41.7–85.4) and 21.2% (95% CI 17.0–26.1), respectively. Young women had greater odds of having a CMD (adjusted odds ratio (aOR) = 5.8, 95% CI 1.8–18.6) and CMDs were associated with living alone (aOR = 3.0, 95% CI 1.1–8.0) and abuse (aOR = 1.5, 95% CI 0.8–2.8).

**Conclusions:**

Pregnant women between 16 and 24 years are at very high risk of mental disorders; services need to target resources for pregnant women under 25, including those in their early 20s. Interventions enhancing social networks, addressing abuse and providing adequate mental health treatment may minimise adverse outcomes for young women and their children.

**Declaration of interest:**

None.

## Background

Young people, defined by the United Nations as between 15 and 24 years, experience a significant burden of disease because of mental disorders globally.^1^ Young women are at particularly high risk; for example, the recent 2017 Mental Health of Children and Young People survey^2^ reported that in the UK, rates for mental disorders in 5–19 year olds were highest among young women aged 17–19 years, with almost a quarter (23.9%) having at least one mental disorder. Another recent UK national survey^3^ reported higher rates of common mental disorders (CMDs), including depression, anxiety disorders, and obsessive–compulsive disorder (OCD) in young women under 25 compared with older women (26% *v.* 17%). The same survey reported rates of post-traumatic stress disorder (PTSD) and self-harm in young women to be 12.6% and 20%, respectively; this was higher than reported in other age groups and in men of the same age, and the rate of self-harm in young women was more than double compared with a previous survey conducted in 2000 using the same methodology.^3^

It is well established that pregnant adolescents are at an increased risk of perinatal depression,^4^ but there has been limited research on pregnant women under 25, or on mental disorders other than depression. As mental disorders in pregnancy are known to be associated with adverse outcomes for both mother and child,^5^ evidence on their prevalence, and associated risk factors, in young women is important in preventing intergenerational transmission of adverse health outcomes and improving public health. Reduction of this public health burden also necessitates addressing risk factors for perinatal mental disorders. Previous research has reported a strong association between good social support and fewer depressive symptoms in perinatal adolescents,^6^^,^^7^ and risk factors include poverty, childhood maltreatment, low self-efficacy and body satisfaction.^4^ However, other factors known to disproportionately affect young people (and not just adolescents), have not been investigated in pregnant young women; for example, young women (<25 years) are more likely to be victims of domestic and sexual violence than older women,^8^ and have high rates of unemployment.^9^

## Aims of our study

We therefore aimed to:

- compare the population prevalence estimates of mental disorders (including depression, anxiety disorders, PTSD, OCD, eating disorders), history of self-harm, and recent thoughts of self-harm, in young pregnant women aged between 16 and 24 years against older women in a representative pregnancy cohort in South-East London;
- compare the correlates of mental disorders in young pregnant women with those in women aged ≥25 years.

We hypothesised that young pregnant women (<25 years old) would be at higher risk of having CMDs (depression, anxiety disorders, PTSD, OCD) than pregnant women aged 25 years or older, and that this would be associated with low levels of social support (living alone or homeless versus living with family or partner), having experienced abuse and unemployment, after adjusting for *a priori* confounders.

## Method

### Study design and population

This paper reports the results of a secondary data analysis of a pregnancy cohort recruited from an inner-city London maternity service, between 10 November 2014 and 30 June 2016. The cohort of pregnant women were recruited using sampling stratified according to responding positively or negatively to two depression screening questions (‘Whooley questions’), which are routinely asked by UK midwives at a woman's first antenatal (or ‘booking’) appointment.^10^ This recruitment method was designed to answer a primary research question on the effectiveness of the Whooley questions (see Howard *et al*,^10^ for details). A random sample of women who were Whooley negative (W−, i.e. answered no to both questions) and all women who were Whooley positive (W+, i.e. answered yes to one of these questions) were invited to participate. Interviews were conducted up to 3 weeks after women's booking appointment. Women were excluded if they: (a) were <16 years; (b) did not respond to the Whooley questions; (c) had undergone maternity booking elsewhere; or (d) had a termination or miscarriage between booking and baseline interview. For full details on study design and population, see Howard *et al*.^10^

### Outcomes

#### Mental disorders

Researchers administered the Structured Clinical Interview for DSM-IV-TR Axis I Disorders (SCID-I-Research Version)^11^ Axis I mood episodes, mood disorders and anxiety disorders module; SCID Axis I eating disorders module (SCID-I) and SCID II personality disorders subsection module for borderline personality disorders^12^ to establish whether women's symptoms met criteria for one or more current mental disorders. CMDs were defined as including anxiety disorders (panic disorder, agoraphobia without panic disorder; social phobia; generalised anxiety disorder), OCD, PTSD and depressive disorders (major depressive disorder, mixed anxiety depression).

#### Other measures

Sociodemographic, obstetric and medical history details were collected at interview. Women who had their first antenatal appointment at ≥13 weeks of pregnancy were defined as ‘late bookers’.^13^ Self-reported ethnic groups were categorised into ‘White’, ‘Black African/Caribbean or Black British’, or ‘Asian/mixed/other ethnic groups’. A proxy measure of poverty was used (educational level – no formal qualifications versus other) as self-reported income was missing for over 20% of the study population; the link between poverty and poor educational outcomes is well established, with multiple studies demonstrating that poverty has a large and persistent influence on educational achievement.^14^^,^^15^

Self-reported immigration status was categorised as ‘secure legal status’ (UK National, European Economic Area citizen or indefinite leave to remain) or ‘insecure legal status’ (temporary admission, exceptional leave to remain, awaiting or appealing initial refusal, spousal/family/ancestral visa). Employment status was categorised: ‘employed, homemaker or student’ or ‘unemployed or unable to work due to disability, illness or immigration status’.

Past year alcohol misuse was assessed using the Alcohol Use Disorders Identification Test (AUDIT),^16^ with a cut-off score of eight for harmful drinking. Past year substance misuse was assessed using the Drug Use Disorders Identification Test (DUDIT), with drug-related problems identified at a cut-off of two.^17^

Measures for current thoughts of self-harm were dichotomised from question ten of the Edinburgh Postnatal Depression Scale (EPDS)^18^ (‘In the past week the thought of harming myself has occurred to me’), using ‘sometimes’ or ‘yes, quite often’ as present, and ‘no, never’ or ‘hardly ever’ as absent, based on previous research into responses associated with suicidality.^19^ Measures for a history of self-harm/attempted suicide were derived from any responses referring to a history within the interview, most commonly arising from the SCID II borderline personality disorder interview question: ‘Have you tried to hurt or kill yourself or ever threatened to do so?’

A composite measure of abuse experienced across the lifetime was used for multivariate analysis; this included domestic abuse (sexual or physical abuse by family member or partner), child abuse and sexual abuse – including attempted or completed. The composite measure was derived from three questionnaires: the modified pregnancy version of the Composite Abuse Scale – Short^20^ (a self-administered questionnaire of sexual, physical and severe abusive partner behaviours in the year before and during pregnancy); the Post-Traumatic Stress Disorder Scale,^21^ which includes questions on rape, attempted rape and non-sexual assault by a known perpetrator (‘being mugged, physically attacked, shot, stabbed, or held at gunpoint’) across the lifetime; and the SCID PTSD module (experiences of traumatic events) across the lifetime.

### Statistical analysis

Analyses were carried out in Stata version 14. Sampling weights were used to account for bias induced by stratified sampling in the study design;^22^ confidence interval estimates were generated from Stata's *svy* command.^23^ Weights were calculated based on the total number of recruited women who were W+ and W− from each age group, out of all those that had appointments at the maternity unit during the study period (see Howard *et al*,^10^ for details). There were 57 women aged <25 years in this cohort, and 488 women aged ≥25 years. Sampling weights specific to each of these age groups were used for all analyses: inverse probability weights for the ‘young women’ group were 198/45 for W+ and 986/12 for W−; for women aged ≥25 years, the probability weights were 694/242 for W+ and 8075/246 for W−.

We used complete case analysis to first examine group differences in sociodemographic and clinical variables in young women compared with women ≥25 years. Second, we examined the association between age and CMDs followed by sensitivity analyses. We examined this association, unadjusted, and then initially adjusted for *a priori* sociodemographic confounders (ethnicity, education level) (model 1), with subsequent models additionally adjusting for employment status (model 2), social support (living status) (model 3) and abuse (model 4). To test for collinearity, chi-squared tests assessed the association between relationship and living status, qualifications and household income level, and unplanned pregnancy and experience of abuse.

#### Missing data and sensitivity analyses

We investigated the patterns of missing CMD data in the adjusted models using chi-squared tests (or Fisher's exact test for cells with *n*<5), by comparing characteristics of included to excluded participants in the final regression model. Two (3.5%) young women, and nine women ≥25 years (1.8%) had missing data for CMD, and a further five women had missing data for sociodemographic or clinical variables (including employment, living status and experience of abuse). To investigate the potential impact of missing data, primarily because of 21 participants declining response to the PTSD module (SCID-I), we conducted a sensitivity analysis to examine the extent to which missing data on PTSD affected the results; first assuming all data missing for PTSD was positive, and second assuming missing data as negative. Because of the small number of young women aged <19 years (*n* = 6), multivariate analyses were also repeated including only women aged 19–24 years.

The research was approved by the National Research Ethics Service, London Committee – Camberwell St Giles (ref no 14/LO/0075).

## Results

### Sociodemographic and clinical characteristics

In total, 545 women participated in the study with a mean age of 32.8 (s.d. = 5.7) years. Of these, 57 women were aged between 16 and 24 years (mean age 21.9, s.d. = 2.0); with only 6 aged under 19. The older age group comprised 488 pregnant women aged 25.1–47.5 years (mean age 34.1, s.d. = 4.6).

Young women in the study population were more likely than women ≥25 years to be Black African/Caribbean or Black British (population prevalence: 63.3% (95% CI 39.0–82.4) *v.* 28.8% (95% CI 23.8–34.3); odds ratio (OR) = 6.9, 95% CI 2.0–24.1, *P* = 0.003; reference category ‘White’); to live alone (population prevalence: 24.2% (95% CI 9.1–50.3) *v.* 9.5% (95% CI 6.6–13.3); OR = 6.6, 95% CI 1.7–25.7, *P* = 0.007; reference category ‘living with partner’); to live with parents (population prevalence: 31.9% (95% CI 13.9–57.4) *v.* 6.0% (95% CI 3.8–9.3); OR = 13.7, 95% CI 3.7–50.5, *P*<0.001) or be homeless/living in emergency accommodation (population prevalence: 10.7% (95% CI 2.8–33.4) *v.* 0.5% (95% CI 0.1–2.2); OR = 54.2, 95% CI 6.3–466.2, *P*<0.001); to be single (population prevalence: 34.1% (95% CI 15.6–59.0) *v.* 7.4% (95% CI 5.0–11.0); OR = 6.4, 95% CI 2.2–19.0, *P* = 0.001); to have an unplanned pregnancy (population prevalence: 65.9% (95% CI 41.0–84.4) *v.* 24.8% (95% 20.2–30.1); OR = 5.9, 95% CI 2.1–16.4, *P* = 0.001); be unemployed/unable to work (population prevalence: 35.0% (95% CI 16.3–59.8) *v.* 9.1% (95% CI 6.3–12.9); OR 5.4, 95% CI 1.9–15.6, *P* = 0.002; reference category ‘employed, homemaker or student’) and to have a low household income (population prevalence: 74.7% (95% CI 31.7–95.0) *v.* 11.9% (95% CI 8.4–16.6); OR = 21.9, 95% CI 3.7–130.5, *P* = 0.001). Young women were also less likely to have other children (population prevalence: 18.7% (95% CI 6.5–43.4) *v.* 53.6% (95% CI 47.8–59.3); OR = 0.2, 95% CI 0.1–0.7, *P* = 0.008) (Table 1). All but 2 of the 16 women who were homeless or living in emergency accommodation had a mental disorder.

**Table 1:** Comparison of sociodemographic and clinical characteristics in young women <25 and women ≥25 years

Characteristic	Women <25 years		Women ≥25 years		OR	95% CI
	Proportion, % (*n*)	Weighted prevalence, % (95% CI)	Proportion, % (*n*)	Weighted prevalence, % (95% CI)		
Ethnicity (*n* = 545)						
White	24.6 (14)	18.3 (6.2–43.2)	55.3 (270)	57.3 (51.5–63.0)	Reference	
Black African/Caribbean or Black British	50.9 (29)	63.3 (39.0–82.4)	30.3 (148)	28.8 (23.8–34.3)	6.9	2.0–24.1
Asian/mixed/other ethnic groups	24.6 (14)	18.3 (6.2–43.2)	14.3 (70)	13.9 (10.4–18.4)	4.1	0.9–19.2
Education/qualifications (*n* = 545)						
No formal qualifications^a^	12.3 (7)	9.2 (2.0–33.9)	3.9 (19)	2.7 (1.4–5.2)	Reference	
GSCE/NVQ level or above	87.7 (50)	90.8 (66.1–98.1)	96.1 (469)	97.3 (94.8–98.6)	0.3	0.05–1.5
Household income (*n* = 416)						
£0–£14 999	74.0 (20)	74.7 (31.7–95.0)	14.7 (57)	11.9 (8.4–16.6)	21.9	3.7–130.5
£14 999–£61 000 or more	26.0 (7)	25.3 (5.0–68.3)	85.4 (332)	88.1 (83.4–91.6)	Reference	
Employment status (*n* = 542)						
Employed/homemaker or student	58.9 (33)	65.1 (40.2–83.8)	88.1 (428)	90.9 (87.1–93.7)	Reference	
Unemployed/unable to work	41.1 (23)	35.0 (16.3–59.8)	11.9 (58)	9.1 (6.3–12.9)	5.4	1.9–15.6
Relationship status (*n* = 545)						
Not single	63.2 (36)	65.9 (41.0–84.4)	89.8 (438)	92.6 (89.0–95.0)	Reference	
Single	36.8 (21)	34.1 (15.6–59.0)	10.2 (50)	7.4 (5.0–11.0)	6.4	2.2–19.0
Living status (*n* = 544)						
Partner	26.3 (15)	31.9 (13.9–57.4)	76.2 (371)	81.9 (77.1–85.8)	Reference	
Parents/family	26.3 (15)	31.9 (13.9–57.4)	7.6 (37)	6.0 (3.8–9.3)	13.7	3.7–50.5
Alone	21.1 (12)	24.2 (9.1–50.3)	12.1 (59)	9.5 (6.6–13.3)	6.6	1.7–25.7
Friends/acquaintance	7.0 (4)	1.5 (0.5–4.4)	3.1 (15)	2.2 (1.0–4.6)	1.7	0.4–7.9
Emergency accommodation/homeless^b^	19.3 (11)	10.7 (2.8–33.4)	1.0 (5)	0.5 (0.1–2.2)	54.2	6.3–466.2
Immigration status (*n* = 545)						
Secure legal status	75.4 (43)	94.8 (89.6–97.5)	85.9 (419)	86.5 (82.0–90.0)	Reference	
Insecure legal status	24.6 (14)	5.2 (2.5–10.4)	14.1 (69)	13.5 (10.0–18.0)	0.4	0.2–0.8
Children						
Has other living children (*n* = 545)	26.3 (15)	18.7 (6.5–43.4)	53.1 (259)	53.6 (47.8–59.3)	0.2	0.1–0.7
Referral to Social Services for this pregnancy (*n* = 543)	14.3 (8)	10.3 (2.3–35.7)	2.3 (11)	1.0 (0.4–2.9)	10.8	1.7–68.5
Obstetric history						
Previous terminations (*n* = 544)	36.8 (21)	47.2 (25.1–70.5)	30.4 (148)	27.7 (22.9–33.2)	2.3	0.9–6.3
Previous miscarriages or still-birth (*n* = 543)	23.6 (13)	24.7 (9.5–50.8)	32.0 (156)	33.1 (27.9–38.8)	0.7	0.2–2.1
Unplanned pregnancy (*n* = 545)	63.2 (36)	65.9 (41.0–84.4)	31.4 (153)	24.8 (20.2–30.1)	5.9	2.1–16.4
Late booking (*n* = 545)	38.6 (22)	21.3 (8.3–44.9)	15.0 (73)	14.3 (10.8–18.9)	1.6	0.5–5.0
Current/ chronic medical conditions (*n* = 544)	43.9 (25)	22.4 (9.1–45.6)	44.4 (216)	42.8 (37.1–48.6)	0.4	0.1–1.1
Current smoking (*n* = 545)	7.0 (4)	8.1 (1.4–35.1)	3.7 (18)	1.6 (0.7–3.6)	5.3	0.8–37.5
History of smoking (*n* = 545)	45.6 (26)	49.1 (26.6–71.9)	46.7 (228)	44.0 (38.4–49.8)	1.2	0.5–3.3
Substance misuse (AUDIT and DUDIT) (*n* = 529)	13.2 (7)	16.0 (4.6–43.0)	12.5 (59)	9.4 (6.6–13.3)	1.8	0.5–7.4

AUDIT, Alcohol Use Disorders Identification Test; DUDIT, Drug Use Disorders Identification Test.

a. In the group of young women of school age (<19 years), only one participant (aged 16.1 years) had not completed a qualification of GCSE level/NVQ or higher; this participant also did not describe themselves as being a student/in education. We were therefore confident to define this participant as having ‘no formal qualifications’.

b. For the adjusted models, this category was combined with ‘friends/acquaintances’ because of the small numbers.

### Lifetime abuse

The population prevalence estimates for lifetime experience of abuse was 38.9% (95% CI 19.3–62.9) in young women and 22.9% (95% CI 18.5–28.1) in women ≥25 years. This included 19.5% (95% CI 7.0– 43.9) of young women and 6.2% (95% CI 4.0–9.7) of women ≥25 years having experienced intimate partner violence. A total of 20.6% (95% CI 7.8–44.4) of young women and 12.3% (95% CI 9.1–16.5) of women ≥25 years had experienced sexual violence; including rape or attempted rape (11.1% (95% CI 3.0–33.5) in young women; 4.6% (95% CI 2.7–7.5) in women ≥25 years). Additionally, 11.2% (95% CI 3.0–33.9) of young women and 5.0% (95% CI 3.0–8.1) of women ≥25 years reported experiencing physical or sexual abuse as a child (under 16 years).

### Mental disorders

The population prevalence estimate for any mental disorder was 67.2% (95% CI 41.7–85.4) in young women compared with 21.2% (95% CI 17.0–26.1) in women ≥25 years (Table 2). For CMDs (depression, anxiety disorders, OCD, PTSD), the population prevalence estimates were 45.1% (95% CI 23.5–68.7) and 15.5% (95% CI 12.0–19.8), respectively. The population prevalence estimates for depressive disorders were 21.7% (95% CI 8.5–45.1) in young women, and 9.2% (95% CI 6.7–12.5) in women ≥25 years. Anxiety disorders were especially prevalent in young women: 25% (95% CI 9.7–50.9) *v.* 6.7% (95% CI 4.5–9.9) in women ≥25 years; particularly social phobia, 22.7% (95% CI 8.1–49.5) *v.* 3.0% (95% CI 1.6–5.7), respectively. The population prevalence of generalised anxiety disorder was 9.6% (95% CI 2.2–33.8) in young women compared with 4.1% (95% CI 2.5–6.6) in women ≥25 years.

**Table 2:** Mental disorders in young women <25 and women ≥25 years

	Women <25 years		Women ≥25 years		OR	95% CI
	Rate % (*n*)	Weighted prevalence, % (95% CI)	Rate % (*n*)	Weighted prevalence, % (95% CI)		
Any SCID diagnosis (*n* = 533)	69.1 (38)	67.2 (41.7–85.4)	38.1 (182)	21.2 (17.0–26.1)	7.6	2.6–21.9
Common mental disorders^a^ (*n* = 534)	58.2 (32)	45.1 (23.5–68.7)	33.0 (158)	15.5 (12.0–19.8)	4.5	1.6–12.2
Depressive disorders (*n* = 545)	40.4 (23)	21.7 (8.5–45.1)	25.4 (124)	9.2 (6.7–12.5)	2.7	0.9–8.3
Major depressive disorder	38.6 (22)	21.3 (8.3–44.9)	23.4 (114)	8.9 (6.4–12.1)	–	–
Mixed anxiety depressive disorder	1.8 (1)	0.4 (0.04–2.9)	2.1 (10)	0.3 (0.2–0.6)	–	–
Anxiety disorder (*n* = 544)	25.0 (14)	25.0 (9.7–50.9)	14.3 (70)	6.7 (4.5–9.9)	4.6	1.4–15.0
Panic disorder	1.8 (1)	0.4 (0.04–2.9)	0.4 (2)	0.2 (0.04–0.4)	–	–
Agoraphobia without panic disorder	1.8 (1)	6.9 (0.9–37.4)	0.4 (2)	0.1 (0.02–0.3)	–	–
Social phobia	14.0 (8)	22.7 (8.1–49.5)	3.7 (18)	3.0 (1.6–5.7)	–	–
Generalised anxiety disorder	14.0 (8)	9.6 (2.2–33.8)	10.7 (52)	4.1 (2.5–6.6)	–	–
Post-traumatic stress disorder^b^ (*n* = 524)	13.2 (7)	2.6 (1.1–6.4)	1.5 (7)	0.6 (0.2–2.2)	–	–
Obsessive–compulsive disorder^b^ (*n* = 545)	5.3 (3)	1.1 (0.3–3.9)	3.3 (16)	2.2 (1.1–4.6)	–	–
Specific phobia^b^ (*n* = 544)	10.5 (6)	21.9 (7.6–49.1)	7.4 (36)	7.7 (5.1–11.4)	–	–
Eating disorder^b^ (*n* = 543)	3.6 (2)	0.8 (0.2–3.3)	2.9 (14)	1.5 (0.6–3.5)	–	–
Borderline personality disorder^b^ (*n* = 543)	5.4 (3)	1.1 (0.3–3.9)	2.1 (10)	0.1 (0.2–2.1)	–	–
Bipolar disorder I and/or II^b^ (*n* = 544)	0	–	0.4 (2)	–	–	–

SCID, Structured Clinical Interview for DSM-IV-TR.

a. Anxiety, depression, post-traumatic stress disorder and obsessive–compulsive disorder.

b. Odds ratio and confidence interval not calculated due to small numbers in each group.

Of the total number of women in the cohort diagnosed with any mental disorders at the research interview, only 13 (34.2%) young women and 47 (25.8%) older women self-reported a problem when asked by the researcher if they have a ‘current mental health issue’. 19.8% (95% CI 7.2– 44.0) of young women, and 6.7% (95% CI 4.4–9.9) of women ≥25 years had a history of self-harm. Also, 3.7% (95% CI 1.7–8.1) of young women and 2.1% (95% CI 1.0–4.2) of women ≥25 years were experiencing current thoughts of self-harm.

### Collinearity between independent variables

There was a strong relationship between living status and relationship status (*P*<0.001), educational qualifications and household income (*P*<0.001), and unplanned pregnancy and experience of abuse (*P*<0.001). We therefore did not include relationship status, household income and unplanned pregnancy in multivariate models.

### Young age and CMDs

In the unadjusted and adjusted models (Table 3) young women had greater odds of having a CMD during early pregnancy than women ≥25 years, even after adjusting for confounders (unadjusted OR = 4.5, 95% CI 1.6–12.2, *P* = 0.004; model 4: adjusted (a)OR = 5.8, 95% CI 1.8–18.6, *P* = 0.003). CMD was associated with living alone (aOR 3.0, 95% CI 1.1–8.0, *P* = 0.026; reference category ‘living with partner’). Lifetime experience of abuse was associated with CMD (aOR = 1.5, 95% CI 0.8–2.8, *P* = 0.251), with a stronger association (aOR = 2.1, 95% CI 1.1–3.9, *P* = 0.020) in the sensitivity analysis assuming missing PTSD data is indicative of PTSD (see below for more details). We did not find evidence that ethnicity, educational level, or unemployment were associated with increased risk of CMDs (‘Black African/ Caribbean or Black British’: aOR = 0.6, 95% CI 0.3–1.2, *P* = 0.154; reference category ‘White’; qualification level: aOR = 0.7, 95% CI 0.1–3.4, *P* = 0.630; employment status: ‘unemployed or unable to work’ aOR = 0.4, 95% CI 0.1–1.3, *P* = 0.145; reference category ‘employed/homemaker or student’).

**Table 3:** Multivariate models of young age and common mental disorders (outcome measure)

Variable	Adjusted model 1 (*n* = 529)			Adjusted model 2 (*n* = 529)			Adjusted model 3 (*n* = 529)			Adjusted model 4 (*n* = 529)		
	OR	95% CI	*P*	OR	95% CI	*P*	OR	95% CI	*P*	OR	95% CI	*P*
Young age	4.8	1.7–13.9	0.003	5.6	2.0–16.0	0.001	5.9	1.9–18.6	0.002	5.8	1.8–18.6	0.003
*Sociodemographic*												
Ethnicity												
White (reference category)	Ref	–	–	Ref	–	–	Ref	–	–	Ref	–	–
Black African/ Caribbean or Black British	0.8	0.4–1.6	0.456	0.8	0.4–1.6	0.518	0.6	0.3–1.2	0.128	0.6	0.3–1.2	0.154
Asian/mixed/other ethnic groups	1.5	0.6–3.4	0.359	1.5	0.7–3.4	0.306	1.5	0.7–3.3	0.330	1.5	0.7–3.4	0.324
Formal qualification	0.9	0.1–7.6	0.898	0.7	0.1–5.0	0.735	0.7	0.1–3.7	0.705	0.7	0.1–3.4	0.630
*Employment status*												
Employed/homemaker/student	Ref	–	–	Ref	–	–	Ref	–	–	Ref	–	–
Unemployed/unable to work	–	–	–	0.5	0.2–1.8	0.316	0.5	0.2–1.5	0.206	0.4	0.1–1.3	0.145
*Social support*												
Living status												
Partner	Ref	–	–	Ref	–	–	Ref	–	–	Ref	–	–
Parents/family	–	–	–	–	–	–	0.7	0.2–3.3	0.654	0.7	0.2–3.3	0.659
Alone	–	–	–	–	–	–	3.2	1.2–8.3	0.020	3.0	1.1–8.0	0.026
Emergency accommodation/ homeless or with friends/ acquaintance	–	–	–	–	–	–	1.7	0.4–7.0	0.431	1.6	0.4–6.4	0.521
*Abuse*												
Lifetime experience of abuse	–	–	–	–	–	–	–	–	–	1.5	0.8–2.8	0.251

### Missing data

Participants excluded from the complete case analysis (*n* = 16) were more likely than those included to have a current chronic physical condition (*P* = 0.019) and to have experienced abuse in their lifetime (*P*<0.001) (supplementary Table 1 available at https://doi.org/10.1192/bjo.2019.6). No other differences between participants' sociodemographic or clinical characteristics were found.

### Sensitivity analyses

The sensitivity analysis that assessed the extent to which missing data affected results by first assuming all missing data on PTSD to be positive (+ve) and then negative (−ve) (adjusted model (*n* = 540): aOR = 5.0, 95% CI 1.5–16.1, *P* = 0.007 and aOR =  6.0, 95% CI 1.9–19.2, *P* = 0.002, respectively), demonstrated comparable results with the complete case analysis (supplementary Table 2). When PTSD missing data was assumed to indicate PTSD, CMDs were more strongly associated with lifetime experience of abuse (aOR = 2.1, 95% CI 1.1–3.9; *P* = 0.020).

As a result of the small number of young women aged <19 years (*n* = 6), analyses were repeated including only women aged 19–24 years. The average age of the young women aged between 19 and 24 years was 22.4 (s.d. = 1.5). Results from the adjusted model closely aligned with the full-cohort analysis (see supplementary Table 3): young women aged 19–24 years had greater odds of having a CMD during pregnancy than women ≥25 years (aOR = 5.7, 95% CI 1.8–18.3, *P* = 0.003, *n* = 522). CMD was associated with living alone (aOR = 2.6; 95% CI 0.9–7.0; *P* = 0.063; reference category ‘living with partner’) and lifetime experience of abuse (aOR = 1.2, 95% CI 0.6–2.5; *P* = 0.526); but was not significant at *P* = 0.05.

## Discussion

### Main findings

Young pregnant women under 25 in this maternity population had a very high prevalence of mental disorders, with population estimates of 67.2% (95% CI 41.7–85.4) compared with 21.2% (95% CI 17.0–26.1) in older women. There was a sixfold increased risk of having a CMD in early pregnancy in young women (aOR =  5.8; 95% CI 1.8–18.6; *P* = 0.003), with a population prevalence of 45.1% (95% CI 23.5–68.7) compared with 15.5% (95% CI 12.0–19.8) in women ≥25 years; prevalence of anxiety disorders were especially high in young women (25%, 95% CI 9.7–50.9), particularly social phobia (22.7%, 95% CI 8.1–49.8). Young pregnant women were also more likely to be Black and minority ethnic, single, live in poverty, homeless or living in emergency accommodation, unemployed or unable to work, and to have an unplanned pregnancy. Risk factors for CMDs included low social support (women with CMDs were more likely to live alone) and being a victim of abuse (strong association in the sensitivity analysis).

### Comparison of our findings with other studies

#### Social support

Low level of social support has previously been found to be a risk factor for depressive symptoms in perinatal adolescents^6^ and adults.^24^ For 16 (i.e. more than one-quarter) of the young women in this cohort, this meant being homeless or living in emergency accommodation. Very little research has been carried out on young women who are homeless and become pregnant, but there is evidence that mental disorders, particularly PTSD and depression, are common among homeless mothers, with many reporting leaving home because of abuse or conflict.^25^ Although we were unable to examine homelessness in more detail, the majority had a mental disorder and had experienced domestic abuse; highlighting the extreme vulnerability of this population.

#### Employment

We hypothesised that unemployment would be a risk factor for CMDs as it has been reported elsewhere that antenatal anxiety and depression are more prevalent in unemployed compared with employed women.^24^ Furthermore, unemployment in 16–24 year olds is 2.5 times the rate for adults in London.^9^ However, although the young pregnant women in this cohort were more likely to be unemployed or unable to work than women ≥25, there was a non-significant but protective effect of unemployment or unable to work for CMDs (aOR =  0.4 95% CI 0.1–1.3, *P* = 0.145). This unexpected result may be explained by the insecure and discriminative nature of employment that is experienced by women who are employed during pregnancy – recent reports show that low paid and insecure work are major problems for young women.^26^ For example, a representative survey of young people in England and Wales, carried out by the Young Women's Trust in 2017, reported that 17% of young women had been paid less than minimum wage, and one in three women had been offered a zero hours contract.^26^ Moreover, a recent large UK survey found that 77% of mothers experienced at least one work-related negative or discriminatory experience because of pregnancy or maternity.^27^ Such experiences included being treated so badly they felt they had to leave their job, being dismissed or receiving a lower pay rise or bonus than their peers. These factors warrant further investigation in a large cohort where the nature of employment and its association with the mental health of pregnant women is a research focus; provision of greater employment security during pregnancy may provide enhanced mental health benefits.

#### Abuse

We found that the rate of lifetime experience of abuse was very high in young pregnant women (population estimate: 38.9%, 95% CI 19.3–62.9), with sexual abuse (20.6%, 95% CI 7.8–44.4) and partner abuse rates (19.5%, 95% CI 7.0–43.9) being particularly concerning; it is important to note that these rates may still underestimate the extent of abuse, as abuse is often underreported.^28^ It has been established in other research that pregnant adolescents have a higher risk of being a victim of violence than older women;^29^ with rates of 7–26% reported elsewhere.^8^^,^^30^ Our reported rates are similar – we found high rates of multiple forms of abuse (intimate partner violence, sexual violence, and physical or sexual abuse experienced as a child) which were elicited by our use of multiple methods of enquiry. These results support the many previous studies finding historic or current abuse to be a strong risk factor for antenatal depression or anxiety disorders.^22^^,^^31^ We found an association between abuse and increased odds of having a CMD, and when addressing the missing data for women who declined to answer the PTSD module questions (by assuming all missing data were positive for PTSD), a stronger association with CMDs was found (aOR =  2.1, 95% CI 1.1–3.9; *P* = 0.020). Pregnant women similarly declining to answer trauma-related questions during clinical interviews could lead to a failure of healthcare professionals to identify symptoms of a mental disorder. Low rates of identification of abuse in pregnant women has been described elsewhere,^32^ but this study highlights how this could also impact on under-identification of PTSD in pregnancy.

### A renewed focus on young women

Young people under the age of 25, whose needs occur while in the transition between health services for children and those for adults, are often neglected by service providers and policymakers. For example, the National Institute for Health and Care Excellence provides specific guidelines for mental disorders such as depression in children and young people, but this does not extend beyond 18 years. Similarly, there are specialist services available for pregnant teenagers but few target the under 25s. However, we demonstrate here that the increased risk for mental disorders is not limited to pregnant teenagers; indeed, only 6 of the 57 young women in this cohort were under the age of 19 (and our sensitivity analysis, which excluded these individuals, demonstrated very similar results to that of the full cohort). Our findings therefore indicate that the vulnerability of young pregnant women highlighted in this paper is not limited to those under 19 years, and is also relevant to those in their early 20s. Although there has been increasing concern about the mental health of young women aged 16–24^33^ there has been little focus, if any, on the even higher rates of these disorders in pregnant women in this age group.

### Implications for research and policy

Our findings suggest that universal services in high-income countries may need to target resources on pregnant women under 25. We also highlight the need to improve identification and interventions for women experiencing abuse, potentially through training of staff and integrated interventions addressing mental health alongside abuse.^34^ Societal and community interventions to address insecure housing, employment and social networks may also reduce the risk of mental disorders.

### Strengths and limitations

This study draws on data from a large and representative sample of women in inner-city London using probability weights to account for the bias introduced by the sampling strategy. A limitation of this study was the relatively small number of young women in this cohort, which led to wide confidence intervals; despite this, population prevalence estimates were derived that demonstrated clear associations between young age (<25 years) and CMDs. However, the study included only six women under the age of 19, which limits conclusions that can be drawn regarding teenage pregnant women (i.e. <19 years); further research, with larger cohorts of young women, would be beneficial.

In conclusion, although there is increasing traction for research and policy focusing on adolescent health^1^^,^^35^ and perinatal mental health^36^ the specific needs of pregnant young women aged between 16 and 24 should also be addressed in healthcare and policy. It is important to invest in health system approaches that focus on young women, their mental health and associated risk factors; this will improve health outcomes for these mothers and the next generation.

## Supplementary material

For supplementary material accompanying this paper visit http://dx.doi.org/10.1192/bjo.2019.6. click here to view supplementary material
