# Supplementary material for: Young pregnant women and risk for mental disorders: findings from an early pregnancy cohort
Source: BJPsych Open. 2019 Mar 7;5(2):e21. doi: 10.1192/bjo.2019.6 (PMC6469235; doi:10.1192/bjo.2019.6)
Supplement: Supplementary file 1 [file S2056472419000061sup001.docx]

## Supplementary Material

**Supplementary Table 1 Characteristics of participants excluded and included from final complete case analysis adjusted model**

|  | **Excluded from model**  (n=16) |  | **Included in model**  (n=529) |  | **p** |
| --- | --- | --- | --- | --- | --- |
| **Socio-demographics** | **n** | **%** | **n** | **%** |  |
| **Age** |  |  |  |  | 0.229 |
| *Women <25 years* | 3 | 5.3 | 54 | 94.7 |  |
| *Women ≥25 years* | 13 | 2.7 | 475 | 97.3 |  |
| **Ethnicity** |  |  |  |  |  |
| *White* | 5 | 1.76 | 279 | 98.2 | 0.123 |
| *Black African/Caribbean or Black British* | 6 | 3.4 | 171 | 96.6 |  |
| *Asian / Mixed / other* | 5 | 6.0 | 79 | 94.0 |  |
| **Employment status ^*^** |  |  |  |  | 0.114 |
| *Employed/homemaker or student* | 9 | 2.0 | 452 | 98.1 |  |
| *Unemployed/ unable to work* | 4 | 4.9 | 77 | 95.1 |  |
| **Relationship status** |  |  |  |  | 0.143 |
| *Not single* | 12 | 2.5 | 462 | 97.5 |  |
| *Single* | 4 | 5.6 | 67 | 94.4 |  |
| **Living status ^*^** |  |  |  |  | 0.155 |
| *Partner* | 8 | 2.1 | 378 | 97.9 |  |
| *Parents/family* | 1 | 1.9 | 51 | 98.1 |  |
| *Alone* | 4 | 5.6 | 67 | 94.4 |  |
| *Hostel/emergency accommodation/homeless or friends/acquaintance* | 2 | 5.7 | 33 | 95.3 |  |
| **Immigration status** |  |  |  |  | 0.080 |
| *Secure legal status* | 11 | 2.4 | 451 | 97.6 |  |
| *Insecure legal status* | 5 | 6.0 | 78 | 94.0 |  |
| **Children** |  |  |  |  |  |
| *Has other living children* | 8 | 2.9 | 266 | 97.1 | 0.982 |
| *Referral to Social Services for this pregnancy* ***^*^*** | 1 | 5.3 | 18 | 94.7 | 0.439 |
| **Obstetric history** |  |  |  |  |  |
| *Previous terminations* | 6 | 3.6 | 163 | 96.5 | 0.572 |
| *Previous miscarriages or still-birth* ***^*^*** | 7 | 4.1 | 162 | 95.9 | 0.268 |
| *Unplanned pregnancy* | 6 | 3.2 | 183 | 96.8 | 0.810 |
| *Late booking* | 4 | 4.2 | 91 | 95.8 | 0.499 |
| **Current/chronic medical conditions** ^*^ | 12 | 5.0 | 229 | 95.0 | 0.019 |
| **Current smoking** | 1 | 4.6 | 21 | 95.5 | 0.488 |
| **History of smoking** | 9 | 3.5 | 245 | 96.5 | 0.432 |
| **Drug or alcohol misuse**^*^ | 1 | 1.5 | 65 | 98.5 | 0.706 |
| **Lifetime experience of abuse (any type)** | 12 | 7.0 | 159 | 93.0 | <0.001 |

* Variables have missing data as described in Table 1.

**Supplementary Table 2: Multi-variate models of young age and common mental disorders: comparing complete case analysis with sensitivity analyses**

|  | **Variable** |  | **Complete case analysis**  (n = 529) | | | **Sensitivity analysis 1 (+ve)**  (n = 540) | | | **Sensitivity analysis 2 (-ve)**  (n = 540) | | |
| --- | --- | --- | --- | --- | --- | --- | --- | --- | --- | --- | --- |
|  |  |  | **OR** | **95%CI** | **p** | **OR** | **95%CI** | **p** | **OR** | **95%CI** | **p** |
|  | **Young Age** |  | **5.8** | **1.8 – 18.6** | **0.003** | 5.0 | 1.5 - 16.1 | 0.007 | 6.0 | 1.9 - 19.2 | 0.002 |
| **Socio-demographic** | **Ethnicity** | *White (reference)* |  |  |  |  |  |  |  |  |  |
|  |  | *Black African/ Caribbean or Black British* | 0.6 | 0.3 -1.2 | 0.154 | 0.7 | 0.3 - 1.4 | 0.292 | 0.5 | 0.3 - 1.2 | 0.122 |
|  |  | *Asian / Mixed / other Ethnic Groups* | 1.5 | 0.7 – 3.4 | 0.324 | 1.9 | 0.9 - 4.1 | 0.093 | 1.4 | 0.6 – 3.0 | 0.444 |
|  | **Formal qualifications** |  | 0.7 | 0.1 – 3.4 | 0.630 | 0.7 | 0.2 – 3.2 | 0.639 | 0.7 | 0.1 – 3.4 | 0.637 |
| **Employment status** |  | *Employed or homemaker (reference)* |  |  |  |  |  |  |  |  |  |
|  |  | *Unemployed/ unable to work* | 0.4 | 0.1 - 1.3 | 0.145 | 0.4 | 0.1 - 1.1 | 0.085 | 0.4 | 0.1 – 1.4 | 0.160 |
| **Social support** | **Living status** | *Partner (reference)* |  |  |  |  |  |  |  |  |  |
|  |  | *Parents/ family* | 0.7 | 0.2 – 3.3 | 0.659 | 0.6 | 0.1 - 2.8 | 0.551 | 0.7 | 0.2 – 3.4 | 0.694 |
|  |  | *Alone* | 3.0 | 1.1 – 8.0 | 0.026 | 2.8 | 1.1 – 7.2 | 0.037 | 3.1 | 1.2 – 8.0 | 0.023 |
|  |  | *Emergency accommodation/ homeless or with friends/ acquaintance* | 1.6 | 0.4 - 6.4 | 0.521 | 1.3 | 0.3 - 5.3 | 0.694 | 1.7 | 0.4 - 6.6 | 0.423 |
| **Abuse** | **Lifetime experience of abuse** |  | 1.5 | 0.8 – 2.8 | 0.251 | 2.1 | 1.1 – 3.9 | 0.020 | 1.3 | 0.7 - 2.5 | 0.423 |

**Supplementary Table 3: Multi-variate models of young age and common mental disorders: comparison between results for young age groups (16-24 years vs 19-24years)**

|  | **Variable** |  | **Complete case analysis**  **(young age: 16-24 years)**  (n = 529) | | | **Sensitivity analysis**  **(young age: 19-24 years)**  (n = 523) | | |
| --- | --- | --- | --- | --- | --- | --- | --- | --- |
|  |  |  | **OR** | **95%CI** | **p** | **OR** | **95%CI** | **p** |
|  | **Young Age** |  | **5.7** | **1.8 – 18.6** | **0.003** | **5.7** | **1.8 – 18.3** | **0.003** |
| **Socio-demographic** | **Ethnicity** | *White (reference)* |  |  |  |  |  |  |
|  |  | *Black African/ Caribbean or Black British* | 0.6 | 0.3 -1.2 | 0.154 | 0.5 | 0.2 - 1.1 | 0.075 |
|  |  | *Asian / Mixed / other Ethnic Groups* | 1.5 | 0.7 – 3.4 | 0.324 | 1.7 | 0.8 - 3.6 | 0.202 |
|  | **Formal qualifications** |  | 0.7 | 0.1 – 3.4 | 0.630 | 0.3 | 0.1 - 1.3 | 0.102 |
| **Employment status** |  | *Employed or homemaker (reference)* |  |  |  |  |  |  |
|  |  | *Unemployed/ unable to work* | 0.4 | 0.1 - 1.3 | 0.145 | 0.4 | 0.1 - 1.1 | 0.076 |
| **Social support** | **Living status** | *Partner (reference)* |  |  |  |  |  |  |
|  |  | *Parents/ family* | 0.7 | 0.2 – 3.3 | 0.659 | 1.1 | 0.3 – 4.5 | 0.845 |
|  |  | *Alone* | 3.0 | 1.1 – 8.0 | 0.026 | 2.6 | 0.9 – 7.0 | 0.063 |
|  |  | *Emergency accommodation/ homeless or with friends/ acquaintance* | 1.6 | 0.4 - 6.4 | 0.521 | 1.7 | 0.5 - 6.4 | 0.430 |
| **Abuse** | **Lifetime experience of abuse** |  | 1.5 | 0.8 – 2.8 | 0.251 | 1.2 | 0.6 - 2.5 | 0.526 |
